# Q&A: 'Toxic' effects of sugar: should we be afraid of fructose?

**DOI:** 10.1186/1741-7007-10-42

**Published:** 2012-05-21

**Authors:** Luc Tappy

**Affiliations:** 1 Department of Physiology, and Service of Endocrinology, Diabetes and Metabolism, Faculty of Biology and Medicine, University of Lausanne, 7 rue du Bugnon, CH-1005 Lausanne Switzerland

## Fructose has recently been the focus of much interest as a possible contributor to the current epidemic of metabolic diseases. What is fructose, and why is it implicated in metabolic disease?

Fructose is a hexose with the same chemical formula, C_6_H_12_O_6_, as glucose. These two sweet-tasting molecules differ structurally, however, as fructose has a keto-group on the first carbon while glucose presents an aldehyde group on the second carbon. Free fructose, together with free glucose, is present in small amounts in fruits and honey. The main part of today's dietary fructose intake comes from sucrose, a disaccharide composed of one molecule of glucose linked to a molecule of fructose through an alpha 1-4 glycoside bond.

The link with metabolic disease is partly circumstantial. Fructose consumption has been low throughout most of human history, but started to increase after the crusades, when Europeans became acquainted with sucrose produced from sugar cane in Asia. It was at first a luxury product, but consumption rapidly increased in the 16th and 17th centuries when sugar became more widely available as a consequence of colonial trading. Its consumption was boosted, first by the introduction of new beverages - tea, coffee, and cocoa in the 17th to 18th centuries; and second with the production of chocolate bars, ice-creams, and sodas at the beginning of the 20th century. Total sugar consumption thus increased from less than 5 kg/person/year in the 1800s to about 40 kg at the turn of the 19th century, and about 70 kg/person/year in 2006. In short, a rapid and continuous increase in consumption has been observed from 1750 until the present day.

In the 1960s, a novel food technology allowed the large-scale, industrial conversion of glucose into fructose. As a result, the US corn industry started preparing what is now known as high fructose corn syrup (HFCS), that is, a concentrated solution of corn-derived glucose and fructose mixed in various relative proportions. Mainly because of its low cost, HFCS consumption replaced approximately one-third of the total sugar consumption in the USA between 1970 and 2000, paralleling to some extent the increasing prevalence of obesity during this period. Consequently, HFCS has been a particular focus of possible blame for the obesity epidemic. However, HFCS consumption has remained very low in other parts of the world where obesity has also increased, and the most commonly used form of HFCS contains about 55% fructose, 42% glucose, and 3% other sugars, and hence is associated with similar total fructose and glucose intakes as with sugar. Furthermore, sucrose is hydrolyzed in the gut and absorbed into the blood as free glucose and fructose, so one would expect HFCS and sucrose to have the same metabolic consequences. In short, there is currently no evidence to support the hypothesis that HFCS makes a significant contribution to metabolic disease independently of the rise in total fructose consumption.

## So why the focus on fructose in particular?

Several reasons. First of all, fructose is not essential for any physiological function that we know of. This is in contrast to glucose, which is used by all cells in the body to generate energy and constitutes the nearly exclusive energy fuel for the brain. As a consequence of this largely exclusive reliance on glucose for brain metabolism, intricate hormonal and neural mechanisms have evolved to maintain a constant level of glucose in the blood.

**Figure 1:**
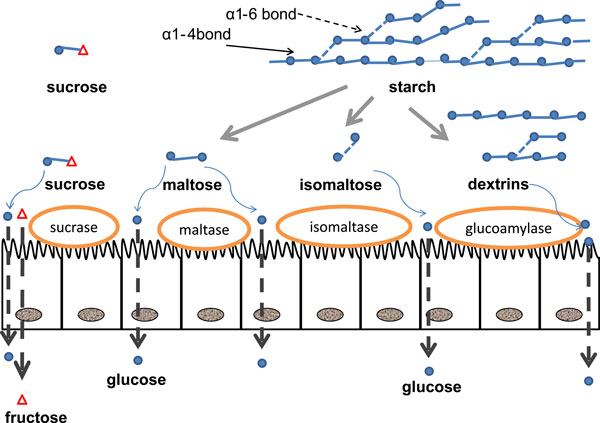
**Digestion and absorption of starch and sugar**. Starch is a polymer of several thousand molecules of glucose, which is digested by the pancreatic enzyme alpha-amylase into maltose, isomaltose, maltotriose (not represented in the figure) and maltodextrins. At the level of the brush border of the intestinal mucosa, specific enzymes generate glucose from maltose (sucrase, maltase), isomaltose (isomaltase) and maltodextrins (glucoamylase). Glucose is then absorbed into the enterocyte by an apical co-transport with NaCl (Sodium-glucose-transporter-1, SGLT1) and transferred to the blood at the basolateral membrane through a facilitated transport mediated by GLUT2. Sucrose is cleaved into glucose and fructose by sucrase at the brush border. Fructose is transported into the enterocyte independently of Na by GLUT5, and due to the presence of fructose metabolizing, gluconeogenic and lipogenic enzymes, part of the absorbed fructose may be metabolized to lactate, glucose, and fatty acids within the enterocytes. Unmetabolized fructose is transferred to the blood at the basolateral membrane by GLUT2.

We do not need to eat sugar to maintain blood glucose levels, however. Until relatively recently, our dietary source of glucose was derived from complex carbohydrates, principally from grains. Grains contain starch, which is a polymer of several thousands of glucose molecules linked together by alpha 1-4 glycosidic bonds, with occasional branching points due to alpha 1-6 glycosidic bonds. Cooked starch can be readily digested by amylase produced by the salivary glands and pancreas, resulting in the formation of maltodextrins (small chains of four to nine glucose molecules), maltose, isomaltose, or triomaltose in the gut lumen (Figure 1). These compounds are subsequently digested into glucose by brush border enzymes of the duodenum and jejunum. Ingestion of starchy products therefore provides a plentiful supply of glucose, which, upon absorption into the circulation, can be used as an energy source by most cells, or be stored as glycogen in the liver and in muscle.

With the exception of a limited amount of free glucose and fructose present in honey and fruits, grains and other starchy food have been the sole source of carbohydrate in the western diet for the major portion of man's history. Sucrose is not only a non-essential dietary element, it has two undesirable consequences. First, because of its rapid digestion, it leads to surges in blood glucose that may place some stress on the homeostatic mechanisms mediated by insulin; and second, it introduces fructose, which we do not need and whose metabolism, when ingested in excessive amounts, imposes an important metabolic burden on the liver.

## How do we metabolize fructose? Is it treated differently from glucose?

Yes it is. Glucose derived from fruits, sugar or digestion of starch is absorbed through the gut into the portal vein. A portion (15 to 30%) of glucose reaching the liver in this way is transported into hepatocytes by the membrane transporter GLUT2. Once in the cell, glucose is converted into glucose-6-phosphate under the control of glucokinase, then into fructose 1-6 diphosphate through the action of phosophofructose kinase and finally to triose-phosphate and pyruvate. Pyruvate can then be decarboxylated to acetyl coenzyme A, and enter the tricarboxylic acid cycle for ATP production. Intracellular ATP and citrate exert a negative feedback on phosphofructokinase, so that hepatic glucose catabolism is tuned to the energy status of the liver cells, and insulin regulates glucokinase expression and the activity of key glycolytic enzymes. Thus, in liver cells, as in other cells of the body, the breakdown of glucose is matched to meet energy requirements.

**Figure 2:**
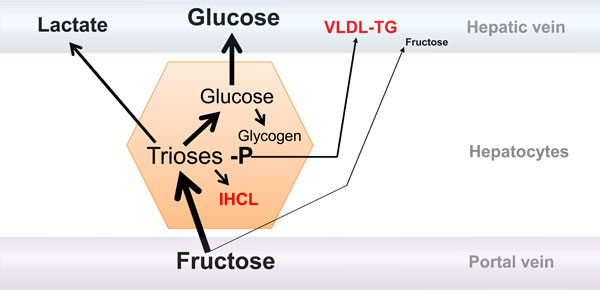
**Metabolism of fructose in the liver**. The majority of fructose in the portal vein is taken up by the liver to be converted into glucose, glycogen, and lactate. A small portion may be either oxidized within hepatocytes or converted into fatty acid, which will be either secreted as very low density lipoprotein-triglyceride (VLDL-TG) particles or stored as intrahepatocellular lipids (IHCL). Only a minor portion escapes liver uptake and reaches the systemic circulation; blood fructose concentrations therefore remain very low even after ingestion of a large fructose load.

By contrast, fructose metabolism is not tuned to energy needs. A limited amount of fructose may be metabolized within the gut enterocytes, but for the most part it is absorbed through the gut into the portal vein. As with glucose, it is transported into hepatocytes by GLUT2. However, once inside the hepatocyte, it is very rapidly converted into fructose-1-phosphate under the action of fructokinase, and then to triose-phosphate under the action of aldolase B. These two enzymes act specifically on fructose and fructose-1-phosphate, respectively, and are regulated neither by insulin nor by the energy status of the cell. As a consequence most fructose in portal blood is rapidly converted into triose-phosphate in hepatocytes. This leads to 1) a high consumption rate of hepatic ATP for the initial phosphorylation of fructose, which can lead, when fructose intake is high, to transient ATP depletion, formation of AMP and degradation of adenosine to uric acid; 2) an overflow of triose-phosphates, which are secondarily converted into lactate or glucose to be released into the circulation; 3) stimulation of glycogen synthesis; and 4) stimulation of the synthesis of fatty acids from the carbons of fructose, through a metabolic pathway known as *de novo *lipogenesis (Figure 2).

## Are there harmful consequences of these features of fructose metabolism?

At a high level of intake, yes, and one of these is increased cardiovascular risk. Paradoxically this in part came to light because of a strong interest, in the 1980s, in the use of pure fructose as a sweetener for type 2 diabetic patients. This was proposed on the grounds that fructose might be less harmful than sucrose or glucose because, unlike glucose, it causes little hyperglycemia after eating (postprandial hyperglygemia), and is metabolized independently of insulin. Furthermore, it enhances energy expenditure compared to similar doses of glucose, which was thought to help prevent weight gain.

However, many short-term studies showed that substituting fructose for starch in the diet of type 2 diabetic patients was associated with an increase in plasma triglyceride concentrations (both fasting and postprandial), raising the possibility that any beneficial effect on glycemic control may be counterbalanced by pro-atherogenic effects of hypertriglyceridemia.

## If everyone's liver cells, not just those of type 2 diabetes patients, make triglycerides, couldn't this also be a hazard for healthy people?

Yes. In healthy subjects, short-term overfeeding studies with large doses of fructose (in the 1.5 to 3 g/kg/day, corresponding to 15 to 30% total energy requirement) have repeatedly reported an increase in fasting and postprandial triglycerides, mainly associated with very low density lipoproteins (VLDLs), and an increase in concentrations of apoB100 (a component of both VLDLs and low-density lipoproteins (LDLs)). Circulating VLDL-triglycerides are significantly associated with cardiovascular disease, so this would indicate increased cardiovascular risk associated with fructose.

Two main mechanisms may account for this effect. First, fructose stimulates hepatic *de novo *lipogenesis, thus contributing additional fatty acids for hepatic triglyceride synthesis, as mentioned earlier. The amount of newly formed fatty acid synthesized from fructose remains small, however. But second, fructose ingestion acutely decreases VLDL-triglyceride (VLDL-TG) clearance in adipose tissue, thus increasing VLDL-TG residence time in the blood. An increase in plasma triglyceride concentration has been generally observed with hypercaloric, high fructose diets, that is, when fructose is associated with excess total energy intake. There is, however, evidence that fructose increases fasting triglyceride even when total energy intake is calculated to match energy requirements.

Moreover, there is strong evidence that 24-hour triglyceride concentration is an independent risk factor for atherosclerosis. In addition, a high plasma VLDL-triglyceride concentration leads to the generation of smaller, more dense LDL particles through the cholesteryl-ester mediated transfer of lipids between VLDL and LDL particles. This process is further enhanced in fructose-induced hypertriglyceridemia, probably because of the impaired VLDL-TG clearance, and hence an increased residence time of VLDL in the blood. Both fructose and sucrose therefore lead to an increased proportion of small dense LDL particles within the LDL fraction, a phenotype that is clearly associated with an increased cardiovascular risk.

In parallel, animal experiments revealed that rodents on a high sucrose or high fructose diet almost invariably develop obesity, insulin resistance and diabetes, dyslipidemia, and even occasionally high blood pressure, the characteristic features of metabolic syndrome, which also together increase the risk of cardiovascular disease. Furthermore, these adverse metabolic effects have been shown to be largely attributable to the fructose component of sucrose. One must recognize, however, that feeding animals a high-fat diet leads to similar metabolic alterations, and that energy excess from any food source may be the critical factor responsible for metabolic alterations.

## If high fructose intake can be responsible for the development of obesity and the associated metabolic disorders that constitute metabolic syndrome, wouldn't this show up in epidemiological studies?

The answer to this question is not straightforward. Several large cohort studies have included a dietary evaluation and a medical follow-up, but their interpretation is problematic, for several reasons. First, until recently, fructose as such did not appear in nutritional databases, and these studies therefore looked at a variety of different variables, some evaluating the effects of calculated total sugar intake, others the effects of calculated fructose intake, while others examined the effects of specific food groups (sugar-sweetened beverages, sweets) that contribute substantially to total fructose intake. Second, the results vary according to how statistical analyses were performed. On one hand, some studies used a statistical analysis that was not adjusted for total energy intake, and documented a positive correlation with obesity. Some of these same studies, however, reported that obesity was associated not only with sugar-sweetened beverages and sweet intakes, but also with the consumption of potatoes and meat. On the other hand, some investigators argued that, in order to conclude that fructose (or sugar) is a major determinant of obesity, it is necessary to establish a positive correlation that is independent of total energy intake. These studies searched for a relationship between obesity and sugar intake expressed as a percentage of total calorie intake and generally failed to observe a significant positive correlation, or even reported a negative correlation. Furthermore, although these studies reported that the incidence of diabetes, dyslipidemia, liver disorders, or high blood pressure correlated positively with sugar intake, these relationships were no longer observed after adjusting for total body weight.

## You say it's hard to distinguish effects of fructose on obesity from effects of any excess eating - could fructose just be encouraging us to eat more?

Yes. Rodents fed *ad libitum *a high-sucrose or a high-fructose diet invariably increase their body weight and body fat mass because of an increased total energy intake. This may be due to a stimulation of sweet receptors in the mouth activating reward pathways within the brain.

Alternatively, ingestion of fructose or sucrose may elicit lower satiety responses than other nutrients. Satiety is a process through which eating sends signals that activate specific brain pathways that in turn regulate appetite. Protein and carbohydrate have long been known to elicit a robust satiety response, mediated in part by an increase in insulin. Some observations suggest that fructose or sugar exert less satiating effects than starch or glucose. Possibly due to a lower insulin response. In humans, there is evidence that a meal containing 30% energy as fructose, compared with a similar meal containing 30% glucose, elicits lower postprandial concentrations of glucose, insulin and leptin, and higher concentrations of ghrelin in the blood. Since high blood glucose, insulin and leptin are known as satiating signals to the brain, while ghrelin stimulates food intake, one would expect that fructose would indeed exert lower satiating effects than other carbohydrates. The significance of this has not been demonstrated in practice, however, and several small studies assessing the satiety induced by meals with various glucose:fructose ratios did not present compelling evidence that fructose and sucrose are less satiating than other foods. A recent meta-analysis quite expectedly demonstrated that fructose intake leads, over short periods, to an increase in body weight when consumed as part of a high-calorie diet, but not as part of an energy balanced diet. This reminds us that body weight is strictly dependent on energy balance, and that, if anything, fructose would increase body weight through an increase in total energy intake.

Obesity is clearly associated with metabolic disease, but not all fat deposits are equal in this respect. Fat stored within the abdominal cavity, that is, visceral fat, is much more closely associated with cardiovascular diseases than subcutaneous fat. It has been proposed, based on one single study, that fructose associated with excess energy intake would preferentially increase visceral fat. This needs to be confirmed in larger, well controlled studies, however.

## What about other aspects of metabolic syndrome? Is fructose implicated in increased fat storage in the liver and for the development of non-alcoholic fatty liver disease?

Overfeeding with 30% energy as fructose nearly doubles intrahepatic fat content in healthy volunteers within a few days. However, overfeeding with lesser amounts of fructose fails to enhance intrahepatic fat significantly, even when exposure is sustained for 4 weeks. Whether fructose exposure of longer duration would lead to continuous, more important deposition of intrahepatic fat and clinical hepatic steatosis (fatty liver) remains presently unknown. No large epidemiological study has evaluated the relationship between fructose or sucrose intake and non-alcoholic fatty liver disease (NAFLD) so far, so the suspicion that fructose may be deleterious for liver cells rests mainly on animal experiments. There are indeed observations, in animal models, that suggest fructose may promote hepatic inflammation and fibrosis, and hence may possibly play a role in the progression of NAFLD to non-alcoholic steatohepatitis (NASH).

## And insulin resistance, could high fructose intake be a cause of this?

Insulin concentration increases after a meal, and is instrumental in maintaining adequate glucose concentrations. It works by stimulating glucose uptake in skeletal muscle and adipose cells, increasing glucose oxidation to generate energy in the form of ATP, and favoring the storage of lipids in adipose tissue. In many obese subjects, and more particularly so in subjects with abdominal obesity, these effects of insulin are blunted, resulting in post-prandial hyperglycemia and hyperlipemia in spite of a normal or even increased insulin secretion. This alteration of insulin's effect, known as insulin resistance, is a major factor responsible for hyperglycemia in type 2 diabetes mellitus, and a prominent feature of metabolic syndrome. The mechanisms remain incompletely understood, but accumulation of triglyceride inside hepatocytes and muscle fibers, generating toxic intracellular lipid metabolites, is known to be involved.

In rodents fed high fructose diets, hyperglycemia and insulin resistance develop, but occur concomitantly with obesity, and hence the effects of fructose *per se *and those linked to excess body fat mass cannot be easily distinguished. There is evidence, however, that hepatic insulin resistance, characterized by increased fasting glucose production and impaired postprandial suppression of glucose output, occurs early after exposure to fructose, before important changes in body composition occur.

In humans, short-term overfeeding with 20 to 30% extra energy provided as fructose leads to a slight increase in fasting plasma glucose, and to a moderate (approximately 10%) increase in fasting glucose production, indicating some impairment of hepatic insulin sensitivity. These changes occur rapidly, within the first week after fructose exposure. There is, however, no detectable decrease in glucose disposal rate induced by insulin when measured by euglycemic hyperinsulinemic clamps (the most reliable method for measuring insulin resistance), indicating no significant whole body insulin resistance. In overweight subjects, fructose overfeeding for 10 weeks led to a modest 1 to 3 kg body weight gain and significantly increased postprandial blood glucose and insulin concentrations, but the average blood glucose concentration barely reached the 2-hour postprandial value of 140 mg/dl, which corresponds to an impaired glucose tolerance. Based on the absence of directly documented insulin resistance, and the modest changes in glycemia and insulinemia observed even after very high fructose intake over several weeks, it appears that fructose *per se *is unlikely to be responsible for important alterations of glucose homeostasis.

**Figure 3:**
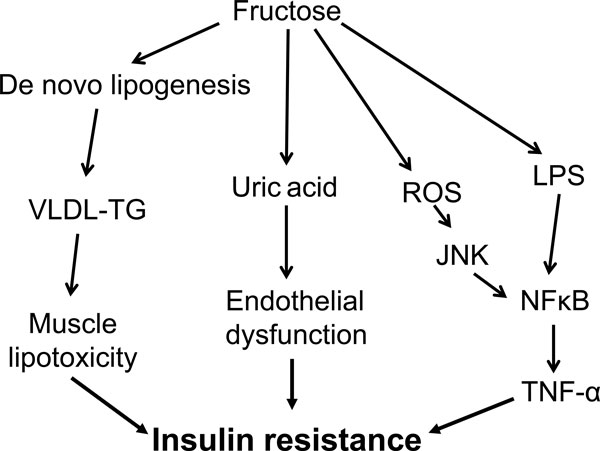
**Putative mechanisms that may link excessive fructose intake to the development of metabolic disorders in the long term**. Stimulation of hepatic *de novo *lipogenesis may lead to the deposition of fat within the liver, which may secondarily be involved in hepatic insulin resistance. Hepatic *de novo *lipogenesis may also cause an increase in VLDL-TG secretion and ectopic deposition of lipids in skeletal muscle, and contribute to muscle insulin resistance through the generation of muscle lipid metabolites. Fructose metabolism in the liver increases uric acid synthesis, and the ensuing hyperuricemia can secondarily be responsible for endothelial cell dysfunction, impaired insulin-induced vasodilation and a consequent failure to increase muscle blood flow after a meal, leading to muscle insulin resistance. In addition, the metabolism of fructose in liver cells can cause the formation of reactive oxygen species (ROS), which can activate nuclear factor (NF)κB, causing inflammation-linked insulin resistance. Finally, fructose can increase the translocation of bacterial endotoxin (lipopolysaccharide (LPS)) into the portal blood, causing endotoxin-mediated stimulation of inflammation. TNF, tumor necrosis factor.

One cannot, however, discard the hypothesis that longer exposure to high fructose intake may be associated with insulin resistance, possibly secondary to increased body fat mass. In addition, a number of mechanisms that could theoretically lead to insulin resistance have emerged from animal or *in vitro *experiments. Specifically, fructose has been shown to cause uric acid-mediated inhibition of endothelium-dependant vasodilation, to impair insulin signaling secondary to oxidative stress, to stimulate hepatic and extra-hepatic inflammation and fibrosis, and to induce lipotoxicity in skeletal muscle (Figure 3). Further studies will be required to evaluate whether these mechanisms may be responsible for the development of insulin resistance in humans with years-long exposure to fructose.

## How much fructose do you have to consume to see adverse effects?

One recent meta-analysis of several small trials in healthy volunteers indicated that fasting and postprandial triglyceride concentrations were increased with intake higher than 100 g and 50 g/day, respectively (corresponding to sucrose intake of 200 and 100 g/day). In an average non-obese individual with moderate physical activity, this corresponds to 15 to 20% and 7.5 to 10%, respectively, of total daily energy intake. Another meta-analysis of studies in which fructose was substituted for starch in the diet of type 2 diabetic subjects indicated that plasma triglyceride concentrations were increased for fructose intakes higher than 60 g/day. However, even with moderate amounts of fructose (40 g/day) that do not change fasting plasma triglycerides, one can observe a shift from large to more atherogenic small, dense LDL particles.

## Is the average consumption of sugar worldwide dangerous?

Consumption of sugar is about 100 to 150 g/day in America, Europe, and Oceania (with important regional differences), corresponding to 50 to 75 g of fructose daily. Since these are averages for the whole population, it means that probably about half of the population has a daily consumption in excess of these figures, and may thus be possibly exposed to fructose-induced dyslipidemia. In the USA, the average consumption of fructose, calculated from the National Health and Nutritional Examination Survey III data, was 55 g/day for the whole population. In adults, however, 10% of the population was consuming more than 15% of their daily energy intake as fructose. Thus, while the major portion of the population may have innocuous fructose intake, a small but still significant portion of the population may be exposed to high, potentially deleterious intakes.

## Is everybody at the same risk of developing dyslipidemia and metabolic diseases from a high fructose intake?

This important question remains unanswered at present, though there are indications that the answer will be 'no'. It is well known that athletes and individuals involved in strenuous physical activity often have high sugar consumption, but as a group have less metabolic and cardiovascular disease than sedentary subjects. A recent study conducted by my laboratory finds that with daily exercise, high fructose consumption does not increase plasma triglyceride concentration. Short-term fructose overfeeding has been shown to cause less dyslipidemia in pre-menopausal women than in men (and no change in hepatic insulin sensitivity). Physical activity, gender, and possibly ethnic or genetic factors may therefore modulate the health effects of fructose. For athletes, a high fructose intake may even be beneficial, as it has been shown that fructose can be metabolized during exercise, and increase performance.

## How might that work?

Athletes frequently use foods and drinks rich in rapidly absorbed carbohydrate during exercise to provide a continuous energy substrate to the working muscle. Lactic acid produced from fructose can be oxidized by the working muscle, and hence moderate amounts of fructose consumed together with glucose during exercise can increase total carbohydrate oxidation and may improve physical performance. Since fructose is known to cause a larger synthesis of hepatic glycogen than glucose, its presence in the diet before and after exercise may also be beneficial to ensure high hepatic glycogen stores.

## On the available evidence, is it time for public health action?

That question cannot be definitively answered on the basis of the available evidence. A high fructose diet, consumed by sedentary individuals, consistently increases hepatic VLDL-TG secretion through stimulation of *de novo *lipogenesis in the liver and decreased extrahepatic VLDL-TG clearance. It also alters LDL particle size, thus leading to alterations of the lipid profile known to be associated with increased cardiovascular diseases. These alterations are, however, observed only at very high levels of fructose intake. In contrast, even at high doses, fructose produces only modest alterations of glucose homeostasis. Fructose indisputably alters hepatic glucose production, but with little impact on blood glucose concentrations, and does not alter whole body insulin sensitivity independently of body weight changes.

But major questions remain to be addressed before we have a clear idea of the role of fructose in metabolic diseases.

## So what do we still need to know?

First, it is not clear whether fructose consumption leads to increased total energy intake and obesity. To address this question further studies focusing on the effects of fructose on food intake control will be needed, and the possibility that fructose may increase energy intake through mechanisms related to addiction will need to be assessed. We also need to assess whether interventions aimed at reducing fructose intake in overweight subjects, by whatever means, will efficiently reduce body weight and cardiovascular and metabolic risk factors. Such studies are obviously needed before implementing litigation or policies aimed at reducing consumption of sugars at the population level.

Second, we do not know whether fructose causes insulin resistance and diabetes mellitus in the long term. Even with very high fructose supplementation, there is only a modest alteration of hepatic glucose metabolism, which may merely represent a metabolic adaptation to the consumption of a glycogenic substrate rather than a step toward diabetes. There are, however, a number of plausible mechanisms documented in animal studies that may lead to deterioration of glucose homeostasis in the long term. We will need more basic and clinical studies to better evaluate whether these data are relevant to human health.

Finally, we need a better understanding of the genetic and environmental factors in the effect of fructose consumption. There is good evidence that pre-menopausal women and physically active males and females may be resistant to the adverse metabolic effects of fructose, and it can by hypothesized that other subgroups of individuals may have enhanced responsiveness and would benefit from a dietary restriction. To address this question, we need comparative studies of fructose's effects in populations at increased risk of developing metabolic diseases, such as offspring of subjects with type 2 diabetes, overweight individuals, insulin-resistant subjects, or ethnic groups with a high incidence of metabolic diseases.

## So what can we conclude?

There is clearly cause for immediate concern regarding potential long-term effects of very high fructose intake in patients with metabolic disorders and in subjects already at risk of developing metabolic disease due to overweight or low physical activity. Given the substantial consumption of fructose in our diet, mainly from sweetened beverages, sweet snacks, and cereal products with added sugar, and the fact that fructose is an entirely dispensable nutrient, it appears sound to limit consumption of sugar as part of any weight loss program and in individuals at high risk of developing metabolic diseases. There is no evidence, however, that fructose is the sole, or even the main factor in the development of these diseases, nor that it is deleterious to everybody, and public health initiatives should therefore broadly focus on the promotion of healthy lifestyles generally, with restriction of both sugar and saturated fat intakes, and consumption of whole grains, fresh fruits and vegetables rather than focusing exclusively on reduction of sugar intake.

## Where can I find out more?

Aeberli I, Gerber PA, Hochuli M, Kohler S, Haile SR, Gouni-Berthold I, Berthold HK, Spinas GA, Berneis K: **Low to moderate sugar-sweetened beverage consumption impairs glucose and lipid metabolism and promotes inflammation in healthy young men: a randomized controlled trial**. *Am J Clin Nutr *2011, **94:**479-485.

Bizeau ME, Pagliassotti MJ: **Hepatic adaptations to sucrose and fructose**. *Metabolism *2005, **54:**1189-1201.

Bray GA, Nielsen SJ, Popkin BM: **Consumption of high-fructose corn syrup in beverages may play a role in the epidemic of obesity**. *Am JClin Nutr *2004, **79:**537-543.

Dolan LC, Potter SM, Burdock GA: **Evidence-based review on the effect of normal dietary consumption of fructose on blood lipids and body weight of overweight and obese individuals**. *Crit Rev Food Sci Nutr *2010, **50:**889-918.

Lim JS, Mietus-Snyder M, Valente A, Schwarz JM, Lustig RH: **The role of fructose in the pathogenesis of NAFLD and the metabolic syndrome**. *Nat Rev Gastroenterol Hepatol *2010, **7:**251-264.

Malik VS, Hu FB: **Sweeteners and risk of obesity and type 2 diabetes: the role of sugar-sweetened beverages**. *Curr Diabetes Rep *2012 [Epub ahead of print].

Mayes PA: **Intermediary metabolism of fructose**. *Am J Clin Nutr *1993, **58(suppl):**754S-765S.

Mozaffarian D, Hao T, Rimm EB, Willett WC, Hu FB: **Changes in diet and lifestyle and longterm weight gain in women and men**. *New Engl J Med *2011, **364:**2392-2404.

Moran TH: **Fructose and satiety**. *J Nutr *2009, **139:**1253S-1256S.

Sievenpiper JL, de Souza RJ, Mirrahimi A, Yu ME, Carleton AJ, Beyene J, Chiavaroli L, Di Buono M, Jenkins AL, Leiter LA, Wolever TM, Kendall CW, Jenkins DJ: **Effect of fructose on body weight in controlled feeding trials: a systematic review and meta-analysis**. *Ann Int Med *2012, **156:**291-304

Stanhope KL, Schwarz JM, Keim NL, Griffen SC, Bremer AA, Graham JL, Hatcher B, Cox CL, Dyachenko A, Zhang W, McGahan JP, Seibert A, Krauss RM, Chiu S, Schaefer EJ, Ai M, Otokozawa S, Nakajima K, Nakano T, Beysen C, Hellerstein MK, Berglund L, Havel PJ: **Consuming fructose-sweetened, not glucose-sweetened, beverages increases visceral adiposity and lipids and decreases insulin sensitivity in overweight/obese humans**. *J Clin Invest *2009, **119:**1322-1334.

Tappy L, Le KA: **Metabolic effects of fructose and the worldwide increase in obesity**. *Physiol Rev *2010, **90:**23-46

Vos MB, Kimmons JE, Gillespie C, Welsh J, Blanck HM: **Dietary fructose consumption among US children and adults: the Third National Health and Nutrition Examination Survey**. *Medscape J Med *2008, **10:**160.

